# Slow Cortical Potential Versus Live Z-score Neurofeedback in Children and Adolescents with ADHD: A Multi-arm Pragmatic Randomized Controlled Trial with Active and Passive Comparators

**DOI:** 10.1007/s10802-021-00858-1

**Published:** 2021-09-03

**Authors:** John Hasslinger, Sven Bölte, Ulf Jonsson

**Affiliations:** 1 Center of Neurodevelopmental Disorders (KIND), Centre for Psychiatry Research, Department of Women’s and Children’s Health, Karolinska Institutet & Child and Adolescent Psychiatry, Stockholm Health Care Services, Region Stockholm, Stockholm, Sweden; 2 Child and Adolescent Psychiatry, Stockholm Health Services, Region Stockholm, Stockholm, Sweden; 3 Curtin Autism Research Group, School of Occupational Therapy, Social Work and Speech Pathology, Curtin University, Perth, WA Australia; 4 Department of Neuroscience, Child and Adolescent Psychiatry, Uppsala University, Uppsala, Sweden

**Keywords:** Neurofeedback, Multi-arm RCT, Slow Cortical Potentials, Live Z-score, Working Memory Training

## Abstract

Neurofeedback (NF) as a treatment for Attention Deficit Hyperactivity Disorder (ADHD) has been evaluated in several trials, but the specificity and generalizability of effects remain unclear. This four-arm randomized controlled trial evaluated the efficacy of Slow Cortical Potential (SCP; standard NF protocol) and Live Z-score (LZS; non-standard NF protocol) delivered in high-frequency format (five sessions per week during five weeks), compared to Working-memory training (WMT; active comparator) and Treatment-as-usual (TAU; passive comparator). N = 202 children/adolescents aged 9 to 17 years with ADHD participated. The primary outcome measure was multi-report (self-, teacher-, and parent-report) ADHD core symptoms on the Conners-3, assessed at baseline, posttreatment, and 6-months follow-up. Data were analyzed using a linear mixed model. Between-group differences were scarce and did not show a distinct pattern. Superiority of LZS over TAU at endpoint were observed for teacher-rated measures only, while significant differences between SCP and TAU were restricted to posttreatment measurements. Contrary to our expectations, LZS outperformed SCP at endpoint for teacher-rated hyperactivity (-5.37; 95% CI: -10.14 to -0.60; p = .028; *d* = *-.36*) and overall ADHD symptoms (-2.20; -4.18 to -0.22; p = .030; *d* = *-.41*). There was no indication that either form of NF was superior to WMT. No severe adverse events were reported during the trial, whereas transient stress-related problems were quite frequent. Overall, the results from this pragmatic trial do not provide convincing support for broad implementation of NF in child and adolescent psychiatric services. Future research should try to clarify for whom and under what circumstances NF might be a viable treatment option.

**Supplementary Information:**

The online version contains supplementary material available at 10.1007/s10802-021-00858-1.

## Background

Attention Deficit and Hyperactivity Disorder (ADHD) is a heritable and disabling neurodevelopmental condition, defined by age-inappropriate patterns of inattention, hyperactivity and impulsivity (Thapar, 2018), and with an estimated world-wide prevalence of 5.3% in childhood (Polanczyk et al., 2014). The condition is characterized by executive dysfunction, low emotional self-control, and motivational challenges (Rubia, 2018). Comorbidity with other neurodevelopmental conditions, mental disorders, and sleep disorders is high (Reale et al., 2017). Left untreated, ADHD may result in a wide range of adverse longer-term outcomes (Bölte et al., 2018), such as hampered academic and occupational careers, impaired social/peer functioning and family conflicts (Barkley et al., 2006; Harpin, 2005; Pingault et al., 2011; Tarver et al., 2014).

Methylphenidate in children and adolescents, and amphetamines in adults, are the preferred first-choice medications for the short-term treatment of ADHD (Cortese et al., 2018). Nonetheless, side effects are common (Sharma & Couture, 2014), and patients may refuse drug treatment or show inconsistent adherence (Brinkman et al., 2018). Long-term effects of pharmacological treatment are poorly investigated, and concerns have been raised regarding height suppression (Swanson et al., 2017) and cardiovascular functioning (Smith et al., 2010), in particular. Such limitations and concerns provide a rationale for the development and evaluation of non-pharmacological interventions, such as neurocognitive training methods (Razoki, 2018).

Neurofeedback (NF) is a non-invasive neurocognitive training method, which aims to improve cortical functioning by training the brain’s electrical activity through operant conditioning principles; thereby, enhancing the brain’s ability for self-regulation, i.e. the flexibility to adapt brain activity to more effectively meet the changing demands of the environment (Arns et al., 2014). Over time, the training might induce neurophysiological changes in the brain (Lévesque et al., 2006) in form of enhanced inhibitory and sustained attention functions and an associated decrease in ADHD core symptoms. Such improvements can be crucial to school performance and positive outcomes in other key activities in the child’s daily life, although the generalization of NF training effects is still unclear. The past decade has seen a steadily growing body of literature concerning NF as a treatment for ADHD symptoms (Bussalb et al., 2019; Cortese et al., 2016; Hodgson et al., 2014; Sonuga-Barke et al., 2013). A meta-analysis by Cortese et al. (2016) found robust, immediate NF effects when analyzing parent ratings for ADHD symptoms (Standardized Mean Differences [SMD] = 0.35, 95% CI = 0.11–0.59), though when analyzing probably blinded ratings (i.e. teachers) the estimated effect size dropped considerably (SMD = 0.15, 95% CI = -0.08–0.38). Van Doren et al. (2018) conducted a meta-analysis of long-term effects ranging from two to twelve months. Compared to passive or semi-active control conditions, they found small effects on inattention at post-treatment (SMD = 0.38, 95% CI = 0.14–0.61), that grew to a medium effect size at follow-up (SMD = 0.57, 95% CI = 0.34–0.81), based on parent ratings. Similarly, the effect size for hyperactivity/impulsivity also increased from post-treatment (SMD = 0.25, 95% CI = 0.05–0.45) to follow-up (SMD = 0.39, 95% CI = 0.19–0.59).

Despite these results, NF has frequently failed to show superiority over active and semi-active comparators, including electromyogram (EMG)-interventions that mimic the active NF set-up (Aggensteiner et al., 2019). When Cortese et al. (Cortese et al., 2016) looked specifically at trials with active and semi-active control conditions (e.g. physical activity, behavioral interventions, cognitive- and attention training, different forms of EMG-feedback), significant differences only remained for hyperactivity/impulsivity for parent ratings (SMD = 0.25, 95% CI = 0.03–0.47). The lack of clear differences is especially striking for so-called sham-NF (e.g. using pre-recorded EEG as feedback source or providing random feedback) (Arnold et al., 2013, 2020; Lansbergen et al., 2011; Schönenberg et al., 2017; Vollebregt et al., 2014), leading some authors to conclude that NF might mainly be a placebo-like intervention (Thibault & Raz, 2017; Thibault et al., 2018). On the other hand, sham-NF studies have been criticized for neglecting important principals of operant conditioning (e.g. using high reward rates, and frequent auto-thresholding), and failing to show that the intended self-modulation has been learned by the participants (Pigott et al., 2018).

Against this background, the clinical usefulness of NF as a broadly implemented treatment for ADHD remains unclear. Several questions of pivotal relevance for real-world practice deserve further investigation. First, and importantly, the available evidence mainly concerns so-called standard protocols, as described by Arns et al. (2014). When only these well researched and established protocols were included in meta-analysis (Cortese et al., 2016), the effects of NF on total ADHD were significant even for blinded raters (SMD = 0.36, 95% CI = 0.04–0.69). Arguably, Slow Cortical Potential NF (SCP) is the most prominent standard NF training protocol in the treatment of ADHD, with multiple trials showing improvements on both inattention and hyperactivity symptoms (Aggensteiner et al., 2019; Gevensleben et al., 2014; Heinrich et al., 2004; Strehl et al., 2017). Apart from ADHD, SCP has also demonstrated promising effects for epilepsy (Strehl et al., 2014; Tan et al., 2009) and migraine (Siniatchkin et al., 2000). Still, so-called non-standard protocols are also widely used in practice. These protocols deviate substantially from standard NF protocols, as they usually combine different protocols or adjust parameters per session or individual participant, and may even include disputed rationales. It has been argued that individualization of protocols may enhance efficacy (Walker & Kozlowski, 2005), and it is therefore important to further clarify the effects of training protocols deviating from standard protocols. Live Z-Score training (LZS) is a popular non-standard protocol due to its uncomplicated implementation. Despite the lack of support from peer-reviewed research (Coben et al., 2019), it is readily used by private practitioners. LZS uses quantitative electroencephalography (qEEG), which compares the EEG activity of an individual to a norm-referenced population (database) by transforming EEG measures to z-scores (Wigton & Krigbaum, 2015). The main feature of LZS is that it uses real-time estimates of these data to provide feedback to the participant during training in an attempt to normalize EEG activity (Collura, 2016). There is considerable variation in LZS regarding parameters used (e.g. amplitude, power or coherence), definition of ranges, and conversion of z-scores into feedback signals (Collura, 2016). To our knowledge, no study has yet compared SCP to LZS.

Second, estimates of the effects of NF relative to other currently used and available cognitive training alternatives are warranted to enable informed decisions on implementation. NF should not be viewed as an alternative to pharmaceutical interventions, but rather as an add-on or complimentary intervention when medication is not a viable option (Lee & Jung, 2017; Pakdaman et al., 2018). The most relevant comparators would therefore be other non-pharmacological interventions, of which Working memory training (WMT) might be the most widely studied and applied one (Cortese et al., 2014). WMT has shown positive effects on working memory (Bergman-Nutley & Klingberg, 2014; Cortese et al., 2014), but meta-analytic findings indicate limited impact on core ADHD symptoms (Cortese et al., 2014; Melby-Lervåg & Hulme, 2013; Sonuga-Barke et al., 2013).

Third, although the learning mechanisms of operant conditioning (e.g. immediate reinforcement) are mostly well understood and implemented in NF (Kamiya, 2011; Sherlin et al., 2011), the optimal number and frequency of NF sessions is unknown. While most NF studies consist of 20 to 40 training sessions and a rate of two to three sessions per week (Cortese et al., 2016; van Doren et al., 2018), neither training frequency, nor intensity has been considered in meta-analyses (Cortese et al., 2016; Sonuga-Barke et al., 2013; van Doren et al., 2018). Only a few studies have implemented high-intensity training. One pilot study used an intensive design consisting of 20 sessions over two weeks, with double sessions daily (Holtmann et al., 2009). Nevertheless, high intensity NF training remains understudied, foremost due to practical reasons (Mayer et al., 2012; Strehl et al., 2017). On the other hand, other neurocognitive training methods, such as WMT are often delivered with higher intensity, with 5 sessions/week being common and also showing higher effects than weekly sessions (Alloway et al., 2013).

Furthermore, it is important to evaluate the ecological validity in trials reflecting the clinical reality of child and adolescent psychiatry. This encompasses ineluctable factors such as common comorbidities (e.g. learning disabilities and ASD), end-user friendly equipment and software, and staff who are trained, but not necessarily experts.

The objective of the present study was to address the above-mentioned research gaps by providing data from a clinical setting. In order to make optimal use of the collected data, the study was designed as a multi-arm randomized controlled trial (Juszczak et al., 2019). We sought to test the following hypotheses:

1. The effect of a high-intensity standard NF protocol (SCP) on ADHD core symptoms is superior to common passive (Treatment as usual [TAU]) and active (WMT) comparators in a clinical setting.
2. The effect of a high-intensity non-standard NF protocol (LZS) on ADHD core symptoms is superior to both passive (TAU) and active (WMT) comparators in a clinical setting.
3. The effect of SCP-NF on ADHD core symptoms is superior to LZS.

## Method

### Trial Design

This study reports primary results from the KITE-trial (clinicaltrials.gov: NCT01841151), a single site, four-arm, randomized controlled pragmatic trial of neurocognitive training interventions in child and adolescent ADHD, conducted at a child and adolescent psychiatric outpatient clinical research unit in Stockholm, Sweden (Hasslinger et al., 2016). Participants were randomly assigned (1:1:1:1) to SCP, LZS, WMT or TAU. Information about the study was disseminated through outpatient facilities, interest organizations and ADHD advocacy groups. Participants were recruited and enrolled continuously between 2013 and 2019, either via self-referral or as clinical referrals via child and adolescent psychiatric and pediatrics clinics predominantly in Stockholm County. Outcome measures (parent ratings, teacher ratings and self-ratings) were collected at baseline, posttreatment and at a 6-month follow-up. This report focuses on effects on ADHD symptoms (primary outcome), executive functions and quality of life (secondary outcomes). The data collection for the KITE-trial also included a range of neuropsychological tests and neurophysiological measures, which will be reported in secondary publications. The main study and several amendments were approved by the Ethical Review Board in Stockholm. Written consent was obtained from all participants and their legal caregivers.

### Participants

Individuals enrolled in the study had received ADHD (ICD-10: F90.0B, F90.0C; DSM- IV-TR: 314.00, 314.01) as their primary diagnosis within the Swedish public healthcare system (Axén et al., 2010), and were 9 to 17 years of age. Exclusion criteria were an IQ < 80 or insufficient Swedish proficiency. Common neurodevelopmental comorbidities such as autism spectrum disorder, learning disabilities and language impairments were not reasons for exclusion. Neither were other comorbid mental disorders, with the exceptions of conditions where treatment was of high priority (e.g. acute depressions, eating disorders) or could change in severity during the course of the study and cause discontinuation (e.g. bipolar disorders, PANS/PANDAS). Ongoing pharmacological treatment of ADHD was allowed, but the dosage had to remain stable during the study. Participants earned points each session, toward a reward gift certificate of SEK 200 (USD ~ 22) that was provided at post assessment. An additional certificate worth SEK 500 (USD ~ 55) was provided after completing follow-up assessments.

### Procedure

Upon informed consent, participants’ medical journals were assessed for inclusion and exclusion criteria by a clinical psychologist. If additional information was needed in order to rule out intellectual disability, a complementary assessment was conducted using Wechsler Intelligence Scale for Children fourth edition or Wechsler Adult Intelligence Scale fourth edition (Wechsler, 2009, 2011). Diagnostic criteria for ADHD were confirmed with the Kiddie Schedule for Affective Disorders and Schizophrenia Interview (Kaufman et al., 1996) with a parent or other caregiver as part of the full-day, baseline assessment. Participants who were medicated with stimulants (methylphenidate or dexamphetamine) had a 48-h wash-out period prior to all assessments. There was no wash-out for non-stimulant medications. All participants were instructed to not change ongoing treatments for ADHD, nor start any new treatments, until follow-up. This included ADHD medication or dosage, the use of weighted blankets, cognitive training games/apps, and dietary supplements. The active conditions (SCP/LZS/WMT) consisted of daily working week sessions (5 sessions/week) during 5 consecutive weeks (25 sessions in total). In the case that a session was missed due to illness or schedule conflicts, such sessions were added at the end, postponing the post-assessment. However, the maximum training period length was seven weeks in order to maintain the high session intensity and for scheduling purposes. All subjects completed at least 23 sessions. The training period was followed by a post assessment within a week after session 25. Two additional booster sessions were conducted shortly before the six-month follow-up assessment. Teacher ratings were sent by mail, while the parent ratings and self-ratings were completed at the clinic during the assessments. Parent forms were sent by mail in case parents were not present during the assessments. Potential adverse effects were tracked via weekly questionnaires during the training period, as well as through spontaneous reports.

### Randomization

The first 100 participants were allocated to their group based on a dual-lane prepared number sequence. One lane included all four groups, and one lane had excluded WMT. The latter was used for participants that previously had conducted WMT in school or at home. A clinical psychologist in the research group allocated participants sequentially to the number sequence, based on the date of their completed application. Once the first 100 participants had been allocated, every new included applicant got their group allocation via random.org, based on the remaining spots per group (i.e., 50 per intervention minus already allocated). The final five participants were randomized simultaneously, in order to avoid predictability.

### Staff and Treatment Fidelity

The interventions were led by 19 trainers (3 clinical psychologist, 3 registered nurses, 1 assistant nurse and 12 students in clinical psychology). However, the number of sessions completed by each trainer varied considerably. All trainers underwent in-house training for all three interventions. Initially, trainers practiced the different interventions on staff members at the clinic and attended sessions lead by experienced trainers. Their initial sessions were supervised by experienced trainers before being permitted to conduct sessions independently. To further ensure consistency, a step-by-step guide for each intervention was developed, and all trainers communicated frequently with each other.

### Interventions

**SCP.** Slow Cortical Potentials are Event Related Potentials that are either electrically negative or positive and last from several hundred msec. to several seconds (Birbaumer, 1999; Gevensleben et al., 2014). They regulate cortical activity and prepare for physical and cognitive actions, in addition to regulating attention and memory (Birbaumer, 1999; Birbaumer et al., 1990; Elbert, 1993). A shift in increased negativity decreases the threshold for neural excitability and increases overall cortical activity (Birbaumer et al., 1990), while a positive shift is associated with decreased excitability and inhibition (Gevensleben et al., 2012).

SCP sessions were conducted with a THERA PRAX-qEEG™ amplifier (neuroConn GmbH, Ilmenau, Germany), using Ag/AgCl electrodes. Impedance was kept under 5 k Ohm. During the task, participants had to steer an object up or down by intentionally creating negative or positive slow cortical potentials. Each trial lasted 10 s.: a 2 s.-baseline calculation and an 8 s. feedback phase. A reward was displayed when the SCP amplitude exceeded ± 40 µV, respectively for 2 s. during the last 4 s. of the trial. The number of so-called transfer trials, where no direct feedback was given except for the potential reward at the end, increased during first three weeks (20% week 1, 40% week 2, 50% week 3–5). Each SCP session consisted of 144 trials split into four blocks (36 trial per block), and lasted around 60 min. Self-regulation success is reported (Ros et al., 2019) and was determined by the ability to differentiate correctly between the conditions during the transfer trials. Analysis was based on the last three seconds of complete blocks from the participants’ last three training sessions. The first block of each session was excluded, to minimize signal drift and to let participants settle in. Participants that correctly generated negative values during the activation trials and positive values during the deactivation trials were defined as learners.

**LZS.** For LZS, we utilized the Atlantis II™ (BrainMaster Ltd, Bedford, Ohio, USA), with AgCl snap connectors. We implemented a 2-channel LZS using the ANI database (Applied Neuroscience Ltd, Florida, USA). The sessions consisted of two blocks with 20 min continuous feedback. Electrode placements were at C3 and C4 for the first block, and Fz and Cz during the second block. Impedance was kept under 5 k Ohm. During the first 5 to 10 min. of each session, feedback was given using BrainCells™ (BrainMaster Ltd.), where the participant has to collect ‘brain cells in a jar’. Thereafter, participants could choose visual stimuli from Netflix™ or Youtube™ on the screen to operationalize the NF. A transparent dimmer window (Tor Ghai, Stockholm, Sweden) was placed on top of the stimuli, which turned dark when the participant’s brain activity deviated too much from the target amplitudes. The targeted Z-score corridor was kept between at ± 1.5 SD, and the threshold was adjusted manually to enable a reward rate of 60–70%. No specific instructions on strategies were provided, and sessions lasted around 60 min.

**WMT.** For WMT, a computerized software program with visuospatial and auditory tasks called Minneslek Flex™ (www.flexprogram.org) was used. It is a training tool that is widely used across Sweden in school settings (Greiff et al., 2012), and is based on the same principles as the well-researched program CogMed™ (Roche & Johnson, 2014). The participants could choose between a Junior and a Senior version that differed on the thematic content while sharing the same structure. In both versions, every session consisted of six different exercises with 12 trials each. The level of difficulty was automatically adjusted based on the participants’ performance. Session length was influenced by the performance, but on average the sessions lasted around 45.

**TAU.** All participants, including the participants randomized to TAU, were instructed to not change ongoing treatments for ADHD, nor start new treatments, until follow-up. No additional restrictions were imposed. Data about ongoing pharmacological treatment were collected, but not for other interventions including dietary supplements. In accordance with regional guidelines for treatment of ADHD, many of the children’s parents underwent psychoeducational parent group-training prior to study inclusion (Axén et al., 2010). No psychological treatments for ADHD were reported.

**Transfer Exercises.** After two weeks of training, participants in all active conditions received so-called transfer cards with images from the respective training modality, which served as a way to transfer the self-regulatory ability drilled during training to everyday situations. Participants were instructed, and reminded in connection with their training sessions, to use these training cards daily as an aid for practicing the respective self-regulation modality at home (e.g. in connection with homework or reading). Parents were instructed to remind the participant.

### Outcome Measures

**Primary.** The ADHD-index and the inattention and hyperactivity/impulsivity subscales of the Swedish full lengths version of the Conners Rating Scales 3^rd^ edition (Thorell et al., 2015) for parent-, teacher- and self-ratings served as primary outcomes. Depending on informant, the Conners-3 full version consists of 99–115 items on a 4-point Likert scale. The ADHD-index is measured by 10 items that are best at discriminating between ADHD and non-ADHD. The maximum score is 20 for parent- and teacher-rating, and 18 for the self-rating version. Inattention also consist of 10 item (parent- and teacher rating; max. score 30) or 11 items (self-rating; max 33), but measures different aspects of inattention and distractibility associated with ADHD. Hyperactivity/impulsivity consists of 14 items (parent- and self-rating, max. score 42) or 18 items (teacher rating, max. score 54), that measure the hyperactivity and impulsivity elements of ADHD. The Swedish Conners-3 version has shown good internal consistency (Cronbach’s alpha: ADHD-index: r = 0.81-0.95; inattention: r = 0.90-0.95; hyperactivity/impulsivity: r = 0.85-97), and the test–retest reliability, measured by the teacher ratings, is also high (r = 0.96-0.99) (Thorell et al., 2018).

**Secondary.** The Behavior Rating Inventory of Executive Functions (BRIEF) (Gioia et al., 2000) was used to assess parent- and teacher-rated executive functions. It consists of 86-items, on a 3-point Likert scale, generating a Global Executive Composite score which consists of a metacognition index and a behavioral regulation index. Metacognition is the ability to cognitively self-manage tasks and is directly related to a child’s ability to problem solve. The behavior regulation index measures the ability to shift cognitive set and modulate emotions and behavior via appropriate inhibitory control. Both indices have shown good internal consistency (Cronbach’s alpha r = 0.96-0.97), and high test–retest reliability (r = 0.80-0.92) (Gioia et al., 2000). The KIDSCREEN-27 (Ravens-Sieberer et al., 2006) is a self-report questionnaire for children aged 8–18 years and was used to assess health-related quality of life (HRQoL). The questionnaire consists of 27 items, of which 10 items constitute the general HRQoL-index. The index provides a global HRQoL-score (ranging from 10 to 50), with good internal consistency (r = 0.82) and high test–retest reliability (r = 0.73). We also included the daily-functioning items from the Conners-3 questionnaires. These items assess the level of impairment related to school-setting, social- and peer relations, and to the home environment. The self-rated and parent versions consist of three items (max score 9), while the teacher version is comprised of two items (max score 6). The daily-functioning items are presented in the online resource (Supplement Table S1).

**Adverse Events.** We tracked adverse events with a comprehensive checklist (Pediatric Side Effects Checklist) covering 47 discomforting problems on 4-point Likert scales, from “no problem” to “highly-problematic/intolerable” (Pavuluri & Janicak, 2004). Caregivers (or participants when deemed appropriate) were asked to fill out the checklist during the assessments, as well as weekly during the intervention period. We focused on newly emerging side effects or side effects that deteriorated from baseline. Adverse events could also be spontaneously reported or observed during the training sessions, but were not documented in a systematic manner.

**Blindness of Outcome Assessors.** At the time of the 6-month follow-up, teachers were asked about their awareness of the students’ study participation via a questionnaire. If they answered yes, we also inquired what intervention they believed that the student was included in (i.e., NF, WMT or control condition), and why they believed so (i.e., information from student or parents, due to behavioral changes, or guessing). It was not feasible to keep the parents blinded, as parents were closely involved and had received thorough information during the application process.

### Statistical Methods

In accordance with the intention-to-treat principles, primary and secondary analyses included all randomized participants for whom data were available at baseline. The number of participants per arm was set in advance at 50, providing a power (1-beta) of > 0.99 for a large effect and 0.80 for a medium effect at alpha = 5% and an expected attrition rate of 10% (G*Power 3.1.7). The originally planned MANOVA was replaced by linear mixed-effects modeling (random regression), which currently is the preferred choice for analysis of repeated-measures data (Gueorguieva & Krystal, 2004). An important advantage of this method is that missing data are handled using maximum likelihood estimation, leading to less biased estimates under the missing at random assumption. The model was specified by using time (baseline, posttreatment, follow-up), treatment group, and the time by group interaction as fixed effects, as well as a random intercept for each participant. A first-order diagonal covariance structure was applied. A separate model was run for each comparison. The results were presented as least-squares means. The treatment effect was expressed as the group difference in the change of least-squares mean raw scores from baseline to posttreatment/follow-up. No adjustments for multiplicity were applied. Student’s t test and Pearson’s chi-squared test were used to determine if the two groups differed at baseline. In case a significant difference was detected, sensitivity analyses were run adjusting for the variable in question. Between-groups effect sizes were estimated by dividing the group difference in the change of least squares mean scores from baseline to posttreatment/follow-up by the pooled standard deviation for the compared groups at baseline. The moderating effect of age on the outcome was explored in sub-analyses. Participants were grouped into children (younger than 13 years) and adolescents (13 years and older). Age by time by group interactions were calculated. When a significant interaction was found, stratified analyses were conducted for children and adolescents separately. Within-group effects from baseline to follow-up were calculated for all four arms using paired-sample t-test. Within-groups effect sizes were calculated by dividing the mean change score with the pooled standard deviations of the two measurements, including adjustment for the correlation of the two measurements. The statistical analyses were designed, supervised and replicated by one of the authors (UJ), who was blinded to the intervention groups. All analyses were conducted using SPSS version 26.

## Results

**Fig. 1:**
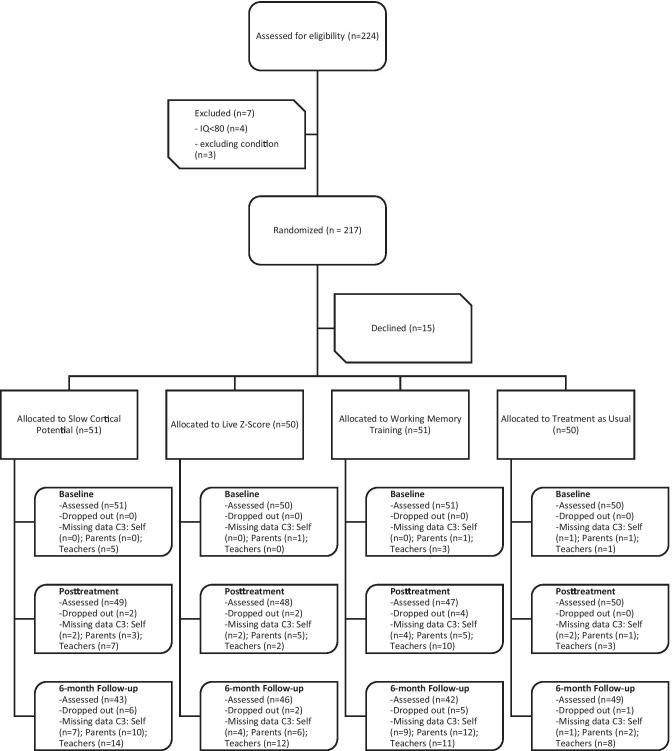
CONSORT Flow Diagram

A total of 224 applicants were evaluated. Seven applicants were excluded due to IQ < 80 or conflicting conditions, while the remaining 217 met the inclusion criteria and were randomized. Fifteen ultimately chose not to participate due to logistic/practical circumstances and/or because they started medication, leaving a total of N = 202 participants. By the posttreatment assessment, eight subjects had dropped-out, while another 14 dropped out before the 6-month follow-up assessment. Thus, 180 subjects completed at least part of the final assessment (see Fig. 1).

The mean age ranged between 12.21 and 12.61 years across the groups, and the male to female ratio was about 3:1. There were no meaningful group differences in IQ, sex, age, nor ADHD severity. Mean scores for parent-ratings were markedly higher (80–83; ≥ 98 percentile) than for teacher-ratings (62–67) and self-ratings (67–72). ASD was somewhat more prevalent (but not significantly different) in the TAU group. The ratio of ADHD sub-types (predominantly hyperactive or combined/ predominantly inattentive) was 3:2 in the NF groups, 4:1 in WMT and almost 1:1 in TAU, but there was only a statistically significant difference between WMT and TAU (*X*^*2*^(1, N = 101) = 6.748, p = 0.009). The use of medication was somewhat lower for SCP, but there was only a statistically significant difference between SCP and TAU (*X*^2^ (1, N = 101) = 4.608, p = 0.032). See Table 1.

**Table 1:** Sample characteristics at baseline by treatment arm

Baseline characteristics	**Slow Cortical Potential** (n = 51)		**Live Z-Score** (n = 50)		**Working Memory Training** (n = 51)		**Treatment as Usual** (n = 50)	
	**M**	**SD**	**M**	**SD**	**M**	**SD**	**M**	**SD**
Age in years	12.35	(2.65)	12.41	(2.30)	12.61	(2.74)	12.21	(2.41)
IQ	104.96	(15.35)	101.80	(12.74)	101.96	(15.87)	100.44	(14.80)
ADHD severity^a^ – Teacher T-score	62.76	(13.37)	65.83	(14.88)	64.27	(15.47)	66.67	(14.61)
ADHD severity^a^ – Parent T-score	80.51	(13.91)	82.84	(9.16)	81.32	(12.85)	82.84	(10.91)
ADHD severity^a^ – Self T-score	69.24	(16.15)	67.12	(15.15)	72.26	(16.67)	70.06	(15.79)
	**n**	**%**	**n**	**%**	**n**	**%**	**n**	**%**
Female	13	25	13	26	9	18	14	28
Teenagers (13y <)	17	33	18	36	22	43	15	30
Comorbid ASD	8^b^	16	7^c^	14	7^d^	14	12^e^	24
Comorbid psychiatric disorder^f^	18	35	15	30	18	35	17	34
Predominantly inattentive^g^	20	39	19	38	11^ h^	22	23^ h^	46
ADHD – medication use	25^i^	49	32	64	33	65	35^i^	70
Melatonin use	6	12	4	8	3	6	7	4

^a^Conners-3 ADHD-index

^b^Childhood Autism (CA) × 1, Atypical autism (AA) × 2, Asperger syndrome (AS) × 5;

^c^AA × 1, AS × 6;

^d^AA × 3, AS × 4;

^e^CA × 3, AA × 4, AS × 5;

^f^Include Mood Disorders, Anxiety disorders, Oppositional Defiant Disorder, Sleeping Disorders, Learning Disorders and Speech Disorders;

^g^ADHD predominantly inattentive subtype. Only one participant in LZS was diagnosed with the predominantly hyperactive subtype, while the rest were diagnosed with the combined subtype

^h^WMT vs. TAU: *X*^*2*^(1, N = 101) = 6.748, p = 0.009;

^i^SCP vs. TAU: *X*^2^ (1, N = 101) = 4.608, p = 0.032

### SCP vs Active and Passive Comparators

On the primary outcome measure, SCP was superior to TAU for inattention at the posttreatment assessment both for the teacher (mean group difference change score: 2.57; 95% CI: 0.45 to 4.69; p = 0.018; Cohen’s *d* = 0.34) and the parent raters (1.78; 0.08 to 3.49; p = 0.041; *d* = 0.31). The parent-rated ADHD-index differed significantly at post (1.68; 0.20 to 3.16; p. = 0.026; *d* = 0.34), but no significant difference remained at follow-up. On the secondary measure, BRIEF, both parent and teacher ratings on the metacognition scale showed a significant difference between SCP and TAU at post (parent: 6.25; 2.43 to 10.06; p = 0.001; *d* = 0.49; teacher: 5.99; 0.04 to 11.94; p = 0.049; *d* = 0.32), as well as at follow-up (parent: 6.31; 1.92 to 10.71; p = 0.005; *d* = 0.49; teacher: 10.99; 2.56 to 19.42; p = 0.011; *d* = 0.58). Sensitivity analysis of the baseline imbalance in medication use did not change the general pattern of results. No significant differences against WMT were found, with the exception of the teacher rating of the ADHD-index at follow-up suggesting that SCP was less effective than WMT (-2.26; -4.35 to -0.18; p = 0.034; *d* = -0.39). No significant group differences were found for any of the self-rated measures (Table 2). For SCP over TAU, a significant age by time by group interaction was found at follow-up on the teacher rated BRI (8.99; 0.44 to 17.54; p = 0.040), with significant effect for children (6.57; 1.46 to 11.68; p = 0.013) but not adolescents (-2.77; -9.81 to 4.28; p = 0.423). Compared to WMT, a significant age by time by group interaction was found for teacher rated hyperactivity at follow-up (8.99; 0.08 to 17.90; p = 0.048), with significant effects for adolescents (-9.72; -17.19 to -2.26; p = 0.012), but not children (-0.92; -6.13 to 4.28; p = 0.724).

**Table 2:** Slow Cortical Potential neurofeedback versus treatment as usual and working memory training from baseline to posttreatment and 6-month follow-up

		Posttreatment			6-month follow-up		
Measure (Rater)	Comparison	Group difference in change score(95% CI)	Sig	Cohen’s *d*	Group difference in change score(95% CI)	Sig	Cohen’s *d*
IN-C3 (T)	vs. TAU	2.57 (0.45 to 4.69)	0.018*	**0.34**	2.64 (-0.05 to 5.32)	0.054	0.35
IN-C3 (P)	vs. TAU	1.78 (0.08 to 3.49)	0.041*	**0.31**	0.92 (-0.85 to 2.69)	0.308	0.16
IN-C3 (S)	vs. TAU	0.83 (-1.17 to 2.82)	0.415	0.12	1.21 (-1.18 to 3.59)	0.319	0.17
HY-C3 (T)	vs. TAU	1.12 (-1.86 to 4.10)	0.459	0.08	0.80 (-3.17 to 4.78)	0.691	0.05
HY-C3 (P)	vs. TAU	1.48 (-0.49 to 3.45)	0.140	0.14	0.84 (-1.76 to 3.43)	0.525	0.08
HY-C3 (S)	vs. TAU	-0.18 (-2.14 to 1.78)	0.858	-0.02	0.01 (-2.18 to 2.20)	0.994	0.00
ADHD-C3 (T)	vs. TAU	1.33 (-0.19 to 2.86)	0.086	0.25	1.13 (-0.89 to 3.15)	0.271	0.21
ADHD-C3 (P)	vs. TAU	1.68 (0.20 to 3.16)	0.026*	**0.34**	1.27 (-0.37 to 2.91)	0.127	0.26
ADHD-C3 (S)	vs. TAU	0.73 (-0.47 to 1.93)	0.229	0.19	0.59 (-0.70 to 1.88)	0.365	0.16
MI-BRIEF (T)	vs. TAU	5.99 (0.04 to 11.94)	0.049*	**0.32**	10.99 (2.56 to 19.42)	0.011*	**0.58**
MI-BRIEF (P)	vs. TAU	6.25 (2.43 to 10.06)	0.001*	**0.49**	6.31 (1.92 to 10.71)	0.005**	**0.49**
BRI-BRIEF (T)	vs. TAU	0.54 (-3.18 to 4.27)	0.773	0.04	3.30 (-1.43 to 8.02)	0.170	0.22
BRI-BRIEF (P)	vs. TAU	1.84 (-0.71 to 4.39)	0.156	0.16	-0.19 (-3.54 to 3.16)	0.911	-0.02
HRQoL-index (S)	vs. TAU	-0.31 (-2.26 to1.64)	0.753	-0.05	1.19 (-0.97 to 3.35)	0.279	0.21
IN-C3 (T)	vs. WMT	0.38 (-1.79 to 2.55)	0.729	0.05	-1.02 (-3.73 to 1.70)	0.461	-0.13
IN-C3 (P)	vs. WMT	0.34 (-1.39 to 2.06)	0.702	0.05	-0.38 (-2.22 to 1.46)	0.683	-0.06
IN-C3 (S)	vs. WMT	0.75 (-1.25 to 2.75)	0.461	0.10	-0.62 (-3.06 to 1.83)	0.619	-0.08
HY-C3 (T)	vs. WMT	-1.95 (-5.00 to 1.11)	0.210	-0.13	-3.72 (-7.74 to 0.30)	0.070	-0.26
HY-C3 (P	vs. WMT	0.41 (-1.59 to 2.41)	0.688	0.04	-1.37 (-4.06 to 1.31)	0.314	-0.12
HY-C3 (S)	vs. WMT	0.74 (-1.23 to 2.71)	0.460	0.08	0.52 (-1.73 to 2.77)	0.647	0.06
ADHD-C3 (T)	vs. WMT	-0.85 (-2.43 to 0.73)	0.289	-0.15	-2.26 (-4.35 to -0.18)	0.034*	**-0.39**
ADHD-C3 (P)	vs. WMT	0.55 ( -0.99 to 2.09)	0.482	0.10	-0.55 (-2.21 to 1.11)	0.511	-0.10
ADHD-C3 (S)	vs. WMT	0.26 (-0.94 to 1.47)	0.665	0.06	-0.29 (-1.60 to 1.03)	0.670	-0.07
MI-BRIEF (T)	vs. WMT	-0.02 (-5.97 to 5.92)	0.993	0.00	3.06 (-5.55 to 11.68)	0.482	0.16
MI-BRIEF (P)	vs. WMT	2.73 (-1.26 to 6.72)	0.179	0.18	3.33 (-1.47 to 8.14)	0.172	0.22
BRI-BRIEF (T)	vs. WMT	0.26 (-3.43 to 3.94)	0.891	0.02	-0.74 (-5.51 to 4.03)	0.759	-0.05
BRI-BRIEF (P)	vs. WMT	0.04 (-2.63 to 2.70)	0.979	0.00	-3.11 (-6.77 to 0.55)	0.095	-0.24
HRQoL-index (S)	vs. WMT	-0.14 (-2.14 to1.87)	0.893	-0.02	0.21 (-2.04 to2.45)	0.857	0.03

Negative numbers favor control condition

*IN-C3* Inattention subscale Conners-3, *HY-C3* Hyperactivity subscale Conners-3, *ADHD-C3* ADHD-index Conners-3, *MI-BRIEF* Metacognition Index BRIEF, *BRI-BRIEF* Behavioral Regulation Index BRIEF, *HRQoL-index* Health-Related Quality of Life index from KIDSCREEN-27, *T* Teacher, *P* Parent, *S* Self

*p <  = 0.05; **p < 0.01

### LZS vs Active and Passive Comparators

Compared to TAU, LZS showed an effect on teacher ratings at follow-up for inattention (3.44; 0.84 to 6.05 p = 0.01; *d* = 0.47), hyperactivity/impulsivity (6.14; 1.97 to 10.31; p = 0.004; *d* = 0.40), metacognition (9.33; 2.07 to 16.60; p = 0.012; *d* = 0.50), and ADHD-index both at posttreatment (2.02; 0.32 to 3.73; p = 0.021; *d* = 0.37) and at follow-up (3.26; 1.21 to 5.30; p = 0.002; *d* = 0.60). On parent ratings, a significant difference was found at posttreatment for the ADHD-index (1.41; 0.02 to 2.81; p = 0.047; *d* = 0.30) and for metacognition (3.80; 0.41 to 7.19; p = 0.028; *d* = 0.30), but did not remain at follow-up. No significant differences were found when comparing LZS to WMT. Overall, no significant group differences were found for any of the self-rating measures (Table 3). No significant age by time by group interaction was found.

**Table 3:** Live Z-Score neurofeedback versus treatment as usual and working memory training from baseline to posttreatment and 6-month follow-up

			Posttreatment			6-month follow-up		
Measure (Rater)		Comparison	Group difference in change score(95% CI)	Sig	Cohen’s *d*	Group difference in change score(95% CI)	Sig	Cohen’s *d*
IN-C3 (T)		vs. TAU	1.27 (-0.65 to 3.18)	0.193	0.17	3.44 (0.84 to 6.05)	0.010*	**0.47**
IN-C3 (P)		vs. TAU	1.13 (-0.56 to 2.83)	0.189	0.20	1.01 (-0.83 to 2.84)	0.280	0.18
IN-C3 (S)		vs. TAU	1.77 (-0.31 to 3.85)	0.095	0.26	1.91 (-0.46 to 4.28)	0.114	0.28
HY-C3 (T)		vs. TAU	2.67 (-0.06 to 5.39)	0.055	0.17	6.14 (1.97 to 10.31)	0.004**	**0.40**
HY-C3 (P)		vs. TAU	1.84 (-0.23 to 3.91)	0.081	0.18	1.24 (-1.32 to 3.80)	0.341	0.12
HY-C3 (S)		vs. TAU	-0.14 (-2.25 to 1.97)	0.896	-0.02	0.79 (-1.53 to 3.11)	0.502	0.10
ADHD-C3 (T)		vs. TAU	2.02 (0.32 to 3.73)	0.021*	**0.37**	3.26 (1.21 to 5.30)	0.002**	**0.60**
ADHD-C3 (P)		vs. TAU	1.41 (0.02 to 2.81)	0.047*	**0.30**	1.77 (-0.01 to 3.54)	0.051	0.37
ADHD-C3 (S)		vs. TAU	0.63 (-0.56 to 1.82)	0.299	0.17	0.71 (-0.57 to 1.96)	0.277	0.20
MI-BRIEF (T)		vs. TAU	3.42 (-1.70 to 8.55)	0.188	0.18	9.33 (2.07 to 16.60)	0.012*	**0.50**
MI-BRIEF (P)		vs. TAU	3.80 (0.41 to 7.19)	0.028*	**0.30**	3.30 (-1.33 to 7.92)	0.161	0.26
BRI-BRIEF (T)		vs. TAU	-0.20 (-3.71 to 3.31)	0.909	-0.01	4.92 (-0.40 to 10.24)	0.070	0.31
BRI-BRIEF (P)		vs. TAU	1.16 (-1.37 to 3.70)	0.366	0.10	1.22 (-2.24 to 4.68)	0.487	0.11
HRQoL-index (S)		vs. TAU	0.92 (-0.80 to2.64)	0.293	0.17	1.50 (-0.39 to3.40)	0.120	0.28
IN-C3 (T)		vs. WMT	-0.91 (-2.87 to 1.05)	0.360	-0.12	-0.22 (-2.86 to 2.41)	0.867	-0.03
IN-C3 (P)		vs. WMT	-0.31 (-2.03 to 1.40)	0.718	-0.05	-0.30 (-2.21 to 1.61)	0.760	-0.05
IN-C3 (S)		vs. WMT	1.67 (-0.43 to 3.75)	0.118	0.24	0.04 (-2.39 to 2.47)	0.976	0.01
HY-C3 (T)		vs. WMT	-0.47 (-3.27 to 2.33)	0.740	-0.03	1.58 (-2.64 to 5.80)	0.461	0.10
HY-C3 (P		vs. WMT	0.77 (-1.33 to 2.87)	0.469	0.07	-1.00 (-3.66 to 1.66)	0.458	-0.09
HY-C3 (S)		vs. WMT	0.71 (-1.41 to 2.83)	0.509	0.08	1.23 (-1.15 to 3.61)	0.308	0.14
ADHD-C3 (T)		vs. WMT	-0.14 (-1.91 to 1.63)	0.875	-0.02	-0.09 (-2.21 to 2.03)	0.932	-0.02
ADHD-C3 (P)		vs. WMT	0.28 (-1.18 to 1.75)	0.700	0.05	-0.03 (-1.85 to 1.79)	0.974	-0.01
ADHD-C3 (S)		vs. WMT	0.14 (-1.05 to 1.34)	0.814	0.04	-0.19 (-1.50 to 1.12)	0.772	-0.05
MI-BRIEF (T)		vs. WMT	-2.55 (-7.67 to 2.57)	0.327	-0.14	1.45 (-5.98 to 8.89)	0.699	0.08
MI-BRIEF (P)		vs. WMT	0.28 (-3.28 to 3.83)	0.877	0.02	0.29 (-4.76 to 5.34)	0.909	0.02
BRI-BRIEF (T)		vs. WMT	-0.69 (-4.16 to 2.77)	0.693	-0.04	0.79 (-4.59 to 6.18)	0.771	0.05
BRI-BRIEF (P)		vs. WMT	-0.65 (-3.30 to 1.99)	0.626	-0.05	-1.72 (-5.49 to 2.06)	0.370	-0.13
HRQoL-index (S)		vs. WMT	1.10 (-0.66 to2.86)	0.218	0.19	0.53 (-1.43 to2.49)	0.595	0.09

Negative numbers favor control condition

*IN-C3* Inattention subscale Conners-3, *HY-C3* Hyperactivity subscale Conners-3, *ADHD-C3* ADHD-index Conners-3, *MI-BRIEF* Metacognition Index BRIEF, *BRI-BRIEF* Behavioral Regulation Index BRIEF, *HRQoL-index* Health-Related Quality of Life index from KIDSCREEN-27, *I* Teacher, *P* Parent, *S* Self

*p <  = 0.05; **p < 0.01

### Head-to-Head Comparison of SCP and LZS

There were no significant differences between SCP and LZS on any primary or secondary measures posttreatment. At follow-up, LZS was superior to SCP on teacher ratings for hyperactivity/impulsivity (-5.37; -10.14 to -0.60; p = 0.028; *d* = -0.36) and ADHD-index (-2.20; -4.18 to -0.22; p = 0.030; *d* = -0.41). See Online resource Table S2 for details. No significant age by time by group interaction was found.

### WMT vs TAU

WMT was superior to TAU on all teacher ratings, both at posttreatment and at follow-up, except for the behavioral regulation index. At follow-up, the parent ratings for overall symptoms was significant (1.79; 0.28 to 3.30; p = 0.020; *d* = 0.35). Metacognition also differed significantly from TAU for the parent rating (3.51; 0.08 to 6.93; p = 0.045; *d* = 0.22), but only at posttreatment (see Table 4). These differences remained largely unchanged after adjustment for imbalance in ADHD-subtype between the groups. A significant age by time by group interaction was found for self-rated hyperactivity at post-treatment (-4.62; -8.60 to -0.64; p = 0.023), with significant effect favoring TAU for children (-2.74; -5.25 to -0.22; p = 0.033), but not for adolescents (1.86; -1.08 to 4.79; p = 0.205).

**Table 4:** Working Memory Training versus Treatment-as-Usual from baseline to posttreatment and 6-month follow-up

	Posttreatment			6-month follow-up		
Measure (Rater)	Group difference in change score(95% CI)	Sig	Cohen’s *d*	Group difference in change score(95% CI)	Sig	Cohen’s *d*
IN-C3 (T)	2.20 (0.04 to 4.36)	0.046*	**0.27**	3.65 (1.01 to 6.30)	0.007**	**0.45**
IN-C3 (P)	1.45 (-0.20 to 3.10)	0.083	0.24	1.28 (-0.42 to 2.99)	0.139	0.21
IN-C3 (S)	0.06 (-1.87 to 2.00)	0.947	0.01	1.81 (-0.53 to 4.14)	0.129	0.25
HY-C3 (T)	3.09 (0.21 to 5.97)	0.036*	**0.20**	4.55 (0.79 to 8.31)	0.018*	**0.30**
HY-C3 (P)	1.07 (-0.86 to 3.00)	0.274	0.10	2.21 (-0.29 to 4.70)	0.083	0.20
HY-C3 (S)	-0.93 (-2.86 to 1.01)	0.344	-0.11	-0.55 (-2.75 to 1.66)	0.625	-0.07
ADHD-C3 (T)	2.17 (0.47 to 3.87)	0.013*	**0.37**	3.37 (1.28 to 5.46)	0.002**	**0.57**
ADHD-C3 (P)	1.16 (-0.14 to 2.47)	0.080	0.23	1.79 (0.28 to 3.30)	0.020*	**0.35**
ADHD-C3 (S)	0.46 (-0.66 to 1.57)	0.421	0.12	0.87 (-0.38 to 2.12)	0.169	0.23
MI-BRIEF (T)	5.88 (0.10 to 11.65)	0.046*	**0.29**	7.82 (0.39 to 15.26)	0.039*	**0.38**
MI-BRIEF (P)	3.51 (0.08 to 6.93)	0.045*	**0.22**	2.84 (-1.16 to 6.83)	0.162	0.18
BRI-BRIEF (T)	0.45 (-2.62 to 3.52)	0.769	0.03	4.14 (-0.51 to 8.78)	0.080	0.26
BRI-BRIEF (P)	1.79 (-0.65 to 4.23)	0.148	0.15	2.90 (-0.50 to 6.29)	0.093	0.24
HRQoL-index (S)	-0.17 (-1.94 to1.60)	0.847	-0.03	0.99 (-0.92 to2.90)	0.308	0.17

Negative numbers favor treatment as usual

*IN-C3* Inattention subscale Conners-3, *HY-C3* Hyperactivity subscale Conners-3, *ADHD-C3* ADHD-index Conners-3, *MI-BRIEF* Metacognition Index BRIEF, *BRI-BRIEF* Behavioral Regulation Index BRIEF, *HRQoL-index* Health-Related Quality of Life index from KIDSCREEN-27, *T* Teacher *P* Parent, *S* Self

*p <  = 0.05; **p < 0.01

### Daily-Functioning

Significant differences were only found for the self-rated items of daily functioning. We found significant effects compared to TAU at post-treatment for both SCP (0.72; 0.04 to 1.39; p = 0.037) and LZS (0.72; 0.18 to 1.27; p = 0.010). WMT showed significant effect compared to TAU at follow-up (0.72; 0.01 to 1.43; p = 0.048).

### Within-Group Differences

We found significant within-group differences over time for all interventions (Online Resource Table S3). For SCP, we found significant differences from baseline to follow-up on five measures at a small to medium effect size (*d* = 0.29–0.65), while LZS showed significant differences for nine of the 14 measures (*d* = 0.26–0.57), and WMT showed significant results for 10 measures (*d* = 0.20–0.53). The highest effect sizes were observed for teacher-rated ADHD-index (LZS: *d* = 0.57; WMT: *d* = 0.53), metacognition (SCP: *d* = 0.55; LZS: *d* = 0.54), and parent metacognition in SCP (*d* = 0.65). In TAU, self-rated hyperactivity/impulsivity (*d* = 0.20) and parent-rated inattention (*d* = 0.22) improved significantly. No changes on HRQoL were noted. Mean scores for complete cases can be found in Online Resource Table S4a-c.

### SCP Self-Regulation

On average, participants differentiated successfully between activation and deactivation trials, and 32 participants differentiated correctly between activation and deactivation (Online Resource Table S5 and Fig. S1). However, only 13 of the 49 (26%) participants were classified as learners. Learners showed higher self-regulation values than non-learners throughout the training period (Online Resource Fig. S1). Significant outcome differences were only found for teacher-rated metacognition (11.03; 1.17 to 20.88; p = 0.029; *d* = 0.69) and behavior regulation (7.93; 0.73 to 15.13; p = 0.032; *d* = 0.57) at posttreatment (Online Resource Table S6).

### Teacher Blindness

In the blindness questionnaire, the majority of teachers (81–97%) were aware of their students’ participation in a research study. However, they were less accurate at correctly pin-pointing the specific intervention the student received (21–66% across intervention arms), even though many had been informed by the students or their caregivers (45–74% across intervention arms). See Online Resource Table S7 for details.

### Adverse Events

No serious or long-lasting adverse events were reported. Nonetheless, a broad array of side effects were reported during the intervention period, with small differences between the groups. Most side effects concerned sleep and tiredness (difficulty falling asleep; sleepiness; fatigue; nightmares), increase in excitability and agitation (agitated; restlessness; irritability), cognition (difficulty concentrating; memory difficulties), mood (anxiety; depression), and headaches (Online resource Table S8). The majority of side effects were reported during the first two weeks. Spontaneously, two subjects reported night-terrors during the first week; however, both subjects had experienced similar issues previously when discontinuing their medication. Headaches were mentioned frequently towards the end of NF sessions, especially for SCP, but they usually stopped once the session was over. Feelings of sleepiness, or even falling asleep, occurred regularly for some subjects receiving SCP, and affected most participants in SCP condition at least at some point.

## Discussion

NF has received considerable research attention in recent years as a non-pharmacological treatment option for ADHD. Our study expands on previous research by investigating the efficacy of both a standard NF protocol (SCP) and a non-standard protocol (LZS) delivered at high-frequency (five sessions/week) in a child and adolescent psychiatric setting. The protocols were compared head-to-head, and against both an active and a passive control condition. Overall, between-groups differences were scarce and did not show a distinct pattern. Furthermore, we did not find any clear indications that treatment effects were moderated by age. Contrary to our expectations, LZS was superior to SCP on a few measures (i.e., teacher-ratings of hyperactivity and overall ADHD-symptoms). While both forms of NF seemed to have some beneficial effects compared to TAU, none of the protocols outperformed WMT on any of the outcome measures.

While all groups receiving an active intervention saw improvements in ADHD core-symptoms over time, meaningful differences between the interventions were few and far between. This aligns well with the lack of clear differences between SCP and active-/semi-active control conditions observed in previous studies (Aggensteiner et al., 2019; Minder et al., 2018), including sham-conditions (Arnold et al., 2020; Schönenberg et al., 2017). The beneficial effects of all active interventions compared to TAU could partly be due to the active components of each intervention, but may also be attributable to non-specific effects (i.e. high level of interaction with trainers) or neuro-suggestion (Thibault et al., 2018). Notably, there were some differences between the two NF protocols when compared to TAU. SCP showed some immediate effects that did not remain at follow-up, while the significant effects of LZS were mainly observed at follow-up. The latter seems to be in keeping with previous findings, suggesting increasing effects of NF on ADHD-symptoms over time (van Doren et al., 2018). Although we did not find any significant differences for self-rated symptoms, all active interventions did show superiority over TAU on the self-rated daily-functioning items, indicating that neurocognitive training may improve functioning beyond symptomatology. To further increase our understanding of the treatment mechanism, in-depth investigations of the participants’ specific NF performances and EEG-markers are needed. Future research should also address subjects’ expectations prior and throughout the interventions, and their impact on self-regulation.

Between-groups differences were mainly observed on teacher-ratings, which is in stark contrast to recent meta-analytic findings (Cortese et al., 2016; Riesco-Matías et al., 2019) where the efficacy of NF on ADHD-symptoms mainly was seen in parent-ratings. The latter has been a major critique of NF, as it suggests that the effects may to a large extent be placebic or based on suggestion (Thibault et al., 2018). However, it has been proposed that the effects are more robust when only looking at standard protocols such as SCP (Cortese et al., 2016; van Doren et al., 2018). Still, the present study does not support this claim, as we could not show lasting effects on ADHD core symptoms for SCP. On the other hand, there were immediate and lasting improvements on metacognition. Further investigation into how NF might improve metacognition is need.

We cannot rule-out that the lack of results in favor for SCP could be due to the relatively high training frequency adapted in this study. Daily training sessions may be too strenuous with little space for consolidation, thereby having a negative impact on the overall process of learning and mastering. Importantly, SCP at high-frequency may be particularly strenuous, as it is more arduous than LZS and WMT owing to its monotonous and repetitious nature, and often leaving subjects confused about how to self-regulate (Hasslinger et al., 2020). LZS and WMT provide more stimulation and less restrictions (e.g. movements), possibly making completion of the task less tedious.

Previous research on LZS protocols is scarce and has focused on 19-channel LZS (Coben et al., 2019). This study is the first large RCT that examines an LZS protocol, implementing a consistent four site (2 × 2 sites) setup. Hence, few comparable studies are available. Groeneveld et al. (Groeneveld et al., 2019) recently showed large positive within-group effects for both adults (Effect size: -1.21) and children (-1.17) on ADHD symptoms using an individualized 4-site LZS protocol. While these effects are substantially higher than observed in the present study (0.37–0.57), the lack of a control conditions limits the validity of such results. We found immediate impacts on overall ADHD symptoms, for both parent and teacher rating. However, at follow-up, significant effects remained only on teacher ratings, including superiority over SCP.

A broad array of side effects was reported during the course of the interventions. While many of these events were deemed unlikely to be causally related to the interventions (the checklist was initially designed for medications), the sheer number of reported events indicates that the training period can be stressful and may temporarily have some negative effects. The most frequently reported events concerned the subjects’ energy levels, impacting their sleep and calmness negatively. Many subjects receiving SCP frequently struggled to stay awake towards the end of the training session. However, the excessive tiredness did not remain once the session was over. These issues still deserve more attention, and more instruments that can capture specific events relevant to NCTs, as these may have an impact on both adherence and outcome.

The modest between-groups effects, and especially the failure to demonstrate superiority compared to WMT, cast doubt on the suitability of NF as a broadly implemented ADHD intervention in a clinical setting. This conclusion is in line with what we currently know about the effectiveness of other non-pharmacological interventions for children and adolescents with ADHD. A recent update on evidence-based treatments classifies several behavioral interventions as well-established treatments, while NF is classified as possibly efficacious and cognitive training as experimental (Evans et al., 2018). Clinical decision making should also be guided by treatment burden, costs and treatment preferences.

Our findings should be viewed in light of some limitations, partly related to the pragmatic nature of this trial. First, the data collection suffers from some missing data, especially for teacher-ratings. It is possible that teachers perceiving greater change in their students were more prone to complete the outcome assessments. While such selection bias might apply equally to all treatment arms, this could make the outcomes more similar across groups. Second, reward limitation might have impeded optimal learning of self-regulation for SCP, as rewards were not connected to performance. Furthermore, the reward was granted at post-assessment, distancing the reward from the training. On the other hand, extensive monetary rewards would probably not be feasible in clinical practice. Third, only 13 (26%) participants in SCP were classified as learners. Also, many participants showed relatively high differentiation values, which may indicate artifact corruption. Fourth, while the sample size was relatively large for an RCT of non-pharmacological treatment for ADHD, it was still quite modest when considering the heterogeneity of the target population. Consequently, undetected relevant differences between the interventions cannot be ruled out. Conversely, the significant differences observed between groups should be interpreted with caution due to the large number of comparisons. Fifth, the use of the default ± 40 µV reward threshold in SCP may have made successful trials too difficult, and hindered optimal learning of self-regulation. Moreover, this may have incited participants to implement physiological strategies, and generate regulation via muscular artefacts (Hasslinger et al., 2020). Sixth, this study focused on symptom measures as outcome, as is common in NF research. However, changes in symptoms might not necessarily transfer into changes in functioning (Bölte et al., 2018), which must be considered when interpreting these findings. Although we included items from the Conners-3 that measure daily-functioning, these measures are limited as they are only compromised of a few items. More robust measures of functioning and impairment, preferably from blinded assessors, should be considered for future studies. Finally, we did not have exact information about the specific content of TAU, which limits generalizability of the findings.

These limitations notwithstanding, the inconsistent results reported here suggest that a more personalized approach to neurocognitive training is needed. Future research should focus on analyzing high and low responders to neurocognitive training to enable clinicians to better predict which children might benefit from these training methods, and for whom the intervention might have little, no, or even predominantly negative effects. For SCP in particular, further emphasis on how to facilitate self-regulation is needed. This may include adjusting number and length of sessions, thresholds, rewards, transfer exercises etc. Furthermore, it is important to gain more knowledge about how neurocognitive training impacts the daily functioning of children and adolescents. In conclusion, our findings do not support NF as a broadly-implemented, standard intervention for ADHD. Future research should focus on analyzing for whom and under what circumstances the intervention might be beneficial.

## Supplementary Information

Below is the link to the electronic supplementary material.

Supplementary file1 (DOCX 229 KB)

## Data Availability

Data are available on request due to privacy or other restrictions.
