# Supplementary material for: Slow Cortical Potential Versus Live Z-score Neurofeedback in Children and Adolescents with ADHD: A Multi-arm Pragmatic Randomized Controlled Trial with Active and Passive Comparators
Source: Res Child Adolesc Psychopathol. 2021 Sep 3;50(4):447–62. doi: 10.1007/s10802-021-00858-1 (PMC8940855; doi:10.1007/s10802-021-00858-1)
Supplement: Supplementary file 1 — Supplementary file1 (DOCX 229 KB) [file 10802_2021_858_MOESM1_ESM.docx]

**Slow Cortical Potential versus Live Z-score neurofeedback in children and adolescents with ADHD: A multi-arm pragmatic randomized controlled trial with active and passive comparators**

John Hasslinger^1,2*^, Sven Bölte^1,2,3^ & Ulf Jonsson^1,2,4^

¹Center of Neurodevelopmental Disorders (KIND), Centre for Psychiatry Research; Department of Women’s and Children’s Health, Karolinska Institutet & Child and Adolescent Psychiatry, Stockholm Health Care Services, Region Stockholm, Stockholm, Sweden.

^2^Child and Adolescent Psychiatry, Stockholm Health Services, Region Stockholm, Stockholm, Sweden.

^3^Curtin Autism Research Group, School of Occupational Therapy, Social Work and Speech Pathology, Curtin University, Perth, Western Australia.

^4^Department of Neuroscience, Child and Adolescent Psychiatry, Uppsala University, Uppsala, Sweden.

^*^ Corresponding author: john.hasslinger@ki.se

| **Supplement Table S1:** Items measuring daily-functioning and impairment, from the Conner’s Rating Scales 3rd edition. | Page 2 |
| --- | --- |
| **Supplement Table S2:** Slow Cortical Potential neurofeedback versus Live Z-score neurofeedback from baseline to posttreatment and 6-month follow-up | Page 2 |
| **Supplement Table S3**: Results of within-group differences baseline to 6-month follow-up. | Page 2 |
| **Supplement Table S4a:** Mean score for teacher-ratings for complete cases per intervention, at baseline, posttreatment and follow-up. | Page 3 |
| **Supplement Table S4b:** Mean score for parent-ratings for complete cases per intervention, at baseline, posttreatment and follow-up. | Page 3 |
| **Supplement Table S4c:** Mean score for self-ratings for complete cases per intervention, at baseline, posttreatment and follow-up. | Page 3 |
| **Supplement Table S5**: Overview of mean differentiation values for of participants last three sessions for feedback trials (mean diff FB) and transfer trials (mean diff TR) for each subject. | Page 4 |
| **Supplement Fig S1:** Self-regulation per session for slow cortical potential neurofeedback. | Page 5 |
| **Supplement Table S6:** Slow Cortical Potential neurofeedback, learners versus non-learners from baseline to posttreatment and 6-month follow-up | Page 5 |
| **Supplement Table S7:** Results for blindness questionnaire for teachers. sorted per intervention. | Page 6 |
| **Supplement Table S8:** Summary of all Adverse Events. according to Pediatric Side Effects Checklist. that increased from baseline to at least problematic. sorted per treatment group. | Page 6 |

| **Supplement Table S1** Items measuring daily-functioning and impairment, from the Conners Rating Scales 3rd edition. | | |
| --- | --- | --- |
| **Rater** | **Item** | **Question** |
| Parent | 106. | Your child’s problems negatively affect schoolwork and grades. |
| Parent | 107. | Your child’s problems negatively affect friend- and family relations. |
| Parent | 108. | Your child’s problems negatively affect the domestic life. |
| Teacher | 112. | The student’s problems negatively affect schoolwork and grades. |
| Teacher | 113. | The student’s problems negatively affect friend- and family relations. |
| Self | 95. | My problems make school really difficult for me. |
| Self | 96. | My problems make it difficult for me to get friends. |
| Self | 97. | My problems make it really difficult for me at home. |
| **Note.** Each item is answered on a 4-point Likert scale, and scored 0-3 per item. | | |

| **Supplement Table S2** Slow Cortical Potential neurofeedback versus Live Z-score neurofeedback from baseline to posttreatment and 6-month follow-up | | | | | | |
| --- | --- | --- | --- | --- | --- | --- |
|  | Posttreatment | | | 6-month follow-up | | |
| Measure (Rater) | Group difference in change score  (95% CI) | Sig. | Cohen’s d | Group difference in change score  (95% CI) | Sig. | Cohen’s d |
| IN-C3 (T) | 0.68 (-1.18 to 2.54) | 0.471 | 0.10 | -0.07 (-2.17 to 2.02) | 0.945 | -0.01 |
| IN-C3 (P) | 1.30 (-0.52 to 3.11) | 0.159 | 0.23 | -0.81 (-3.49 to 1.87) | 0.549 | -0.14 |
| IN-C3 (S) | -0.94 (-3.23 to 1.35) | 0.419 | -0.14 | -0.64 (-3.19 to 1.90) | 0.619 | -0.09 |
| HY-C3 (T) | -1.59 (-4.54 to 1.38) | 0.292 | -0.11 | -5.37 (-10.14 to -0.60) | **0.028*** | **-0.36** |
| HY-C3 (P) | -0.36 (-2.60 to 1.89) | 0.754 | -0.03 | -0.35 (-3.25 to 2.55) | 0.813 | -0.03 |
| HY-C3 (S) | -0.01 (-2.27 to 2.25) | 0.991 | 0.00 | -0.74 (-3.19 to 1.71) | 0.549 | -0.08 |
| ADHD-C3 (T) | -0.71 (-2.26 to 0.85) | 0.367 | -0.13 | -2.20 (-4.18 to -0.22) | **0.030*** | **-0.41** |
| ADHD-C3 (P) | 0.26 (-1.35 to 1.87) | 0.747 | 0.05 | -0.50 (-2.42 to 1.43) | 0.608 | -0.10 |
| ADHD-C3 (S) | 0.12 (-1.30 to 1.54) | 0.867 | 0.03 | -0.06 (-1.45 to 1.34) | 0.935 | -0.02 |
| MI-BRIEF (T) | 2.61 (-2.52 to 7.73) | 0.314 | 0.15 | 1.70 (-7.11 to 10.51) | 0.701 | 0.10 |
| MI-BRIEF (P) | 2.41 (-1.81 to 6.63) | 0.260 | 0.21 | 3.12 (-2.71 to 8.96) | 0.291 | 0.27 |
| BRI-BRIEF (T) | 0.66 (-3.82 to 5.15) | 0.769 | 0.04 | -2.03 (-7.93 to 3.87) | 0.495 | -0.13 |
| BRI-BRIEF (P) | 0.66 (-2.27 to 3.58) | 0.656 | 0.05 | -1.42 (-5.35 to 2.51) | 0.475 | -0.12 |
| HRQoL-index (S) | -1.23 (-3.19 to0.73) | 0.215 | -0.22 | -0.26 (-2.47 to1.95) | 0.814 | -0.05 |
| **Note**: Negative numbers favor Live Z-score.  IN-C3 = Inattention subscale Conners-3; HY-C3 = Hyperactivity subscale Conners-3; ADHD-C3 = ADHD-index Conners-3; MI-BRIEF = Metacognition Index BRIEF; BRI-BRIEF = Behavioral Regulation Index BRIEF; HRQoL-index = Health-Related Quality of Life index from KIDSCREEN-27; T = Teacher; P = Parent; S = Self; * p. <=0.05; ** p.<0.01 | | | | | | |

| **Supplement Table S3** Within-group differences from baseline to 6-month follow-up. | | | | | | | | |
| --- | --- | --- | --- | --- | --- | --- | --- | --- |
| **Slow Cortical Potential** | | | |  | **Live Z-Score** | | | |
| **Measure (Rater)** | **Mean change score**  **(CI 95%)** | **p-value** | **Cohen’s *d*** |  | **Measure** | **Mean change score**  **(CI 95%)** | **p-value** | **Cohen’s *d*** |
| IN-T | 2.24 (0.10 to 4.38) | **0.041** | **0.37** |  | IN-T | 3.02 (1.09 to 4.95) | **0.003** | **0.52** |
| IN-P | 2.41 (0.89 to 3.93) | **0.003** | **0.50** |  | IN-P | 2.50 (0.83 to 4.17) | **0.004** | **0.46** |
| IN-S | 1.80 (-0.22 to 3.81) | 0.08 | 0.27 |  | IN-S | 2.46 (0.58 to 4.34) | **0.012** | **0.39** |
| HY-T | 0.83 (-2.94 to 4.60) | 0.658 | 0.08 |  | HY-T | 6.35 (2.68 to 10.02) | **0.001** | **0.58** |
| HY-P | 1.52 (-0.71 to 3.75) | 0.177 | 0.22 |  | HY-P | 1.98 (-0.11 to 4.06) | 0.062 | 0.29 |
| HY-S | 1.26 (-0.49 to 3.00) | 0.153 | 0.22 |  | HY-S | 2.20 (0.27 to 4.13) | **0.027** | **0.34** |
| ADHD-index-T | 1.32 (-0.28 to 2.93) | 0.104 | 0.29 |  | ADHD-index-T | 3.37 (1.78 to 4.95) | **<0.001** | **0.73** |
| ADHD-index-P | 1.81 (0.48 to 3.14) | **0.009** | **0.43** |  | ADHD-index-P | 2.26 (0.75 to 3.78) | **0.004** | **0.46** |
| ADHD-index-S | 1.21 (0.12 to 2.29) | **0.030** | **0.35** |  | ADHD-index-S | 1.37 (0.26 to 2.47) | **0.016** | **0.38** |
| MI-T | 9.52 (0.91 to 18.13) | **0.032** | **0.47** |  | MI-T | 9.08 (3.65 to14.51) | **0.002** | **0.62** |
| MI-P | 7.74 (3.37 to 12.11) | **0.001** | **0.61** |  | MI-P | 4.65 (-0.35 to 9.65) | 0.067 | 0.31 |
| BRI-T | 3.16 (-0.33 to 6.65) | 0.074 | 0.38 |  | BRI-T | 4.80 (-0.55 to10.16) | 0.077 | 0.32 |
| BRI-P | 1.50 (-1.42 to 4.42) | 0.305 | 0.17 |  | BRI-P | 2.97 (-0.31 to 6.26) | 0.075 | 0.30 |
| HRQoL-index-S^a^ | -0.56 (-2.43 to 1.32) | 0.551 | -0.09 |  | HRQoL-index-S^a^ | -1.15 (-2.61 to 0.31) | 0.119 | -0.23 |
| **Working Memory Training** | | | |  | **Treatment-as-Usual** | | | |
| **Measure** | **Mean change score**  **(CI 95%)** | **p-value** | **Cohen’s *d*** |  | **Measure** | **Mean change score**  **(CI 95%)** | **p-value** | **Cohen’s *d*** |
| IN-T | 2.87 (0.72 to 5.02) | **0.010** | **0.43** |  | IN-T | -0.79 (-2.75 to 1.17) | 0.420 | -0.13 |
| IN-P | 2.37 (0.93 to 3.82) | **0.002** | **0.55** |  | IN-P | 1.22 (0.14 to 2.31) | **0.028** | **0.33** |
| IN-S | 2.18 (0.49 to 3.88) | **0.013** | **0.40** |  | IN-S | 0.77 (-0.92 to 2.46) | 0.363 | 0.13 |
| HY-T | 4.47 (1.49 to 7.45) | **0.004** | **0.48** |  | HY-T | -0.56 (-3.27 to 2.15) | 0.677 | -0.07 |
| HY-P | 2.58 (0.41 to 4.74) | **0.021** | **0.39** |  | HY-P | 0.31 (-1.37 to 1.99) | 0.713 | 0.05 |
| HY-S | 0.57 (-1.36 to 2.50) | 0.552 | 0.09 |  | HY-S | 1.43 (0.02 to 2.84) | **0.048** | **0.29** |
| ADHD-index-T | 2.80 (1.06 to 4.54) | **0.002** | **0.55** |  | ADHD-index-T | -0.66 (-2.12 to 0.79) | 0.362 | -0.15 |
| ADHD-index-P | 2.22 (1.07 to 3.37) | **0.000** | **0.63** |  | ADHD-index-P | 0.60 (-0.47 to 1.67) | 0.266 | 0.16 |
| ADHD-index-S | 1.44 (0.53 to 2.36) | **0.003** | **0.50** |  | ADHD-index-S | 0.78 (-0.13 to 1.70) | 0.090 | 0.25 |
| MI-T | 4.38 (-2.21 to 10.98) | 0.185 | 0.25 |  | MI-T | -1.62 (-6.43 to 3.19) | 0.498 | -0.12 |
| MI-P | 3.50 (0.18 to 6.82) | **0.040** | **0.39** |  | MI-P | 1.11 (-1.45 to 3.66) | 0.387 | 0.13 |
| BRI-T | 2.65 (-1.35 to 6.66) | 0.187 | 0.24 |  | BRI-T | -0.99 (-3.96 to 1.99) | 0.505 | -0.12 |
| BRI-P | 3.97 (1.29 to 6.65) | **0.005** | **0.54** |  | BRI-P | 1.39 (-1.02 to 3.79) | 0.253 | 0.17 |
| HRQoL-index-S^a^ | -0.80 (-2.36 to 0.76) | 0.305 | -0.16 |  | HRQoL-index-S^a^ | 0.37 (-1.01 to 1.76) | 0.592 | 0.08 |
| **Note**. ^a^ Negative number indicates improvement  SCP = Slow Cortical Potential; LZS = Live Z-Score; WMT = Working Memory Training; TAU = Treatment-as-Usual; IN = Inattention scale; HY = Hyperactivity/impulsivity scale; BRI = Behavior Regulation Index; MI = Metacognition Index; HRQoL-index = Health-Related Quality of Life index from KIDSCREEN-27; S = Self; P = Parent; T = Teacher | | | | | | | | |

| **Supplement Table S4a** Mean score for teacher-ratings for complete cases per intervention, at baseline, posttreatment  and follow-up | | | | | | | | | |
| --- | --- | --- | --- | --- | --- | --- | --- | --- | --- |
|  |  | **Slow Cortical**  **Potential** | | **Live Z-Score** | | **Working Memory**  **Training** | | **Treatment as Usual** | |
| **Measure** | **Time** | **Mean (SD)** | **n** | **Mean (SD)** | **n** | **Mean (SD)** | **n** | **Mean (SD)** | **n** |
| **IN-C3** | Baseline | 16.29 (7.34) | 46 | 16.15 (6.98) | 50 | 16.58 (8.48) | 48 | 16.54 (7.69) | 49 |
| **IN-C3** | Post | 14.49 (7.32) | 44 | 15.19 (7.11) | 48 | 14.13 (7.65) | 41 | 16.34 (8.23) | 47 |
| **IN-C3** | FU | 15.93 (7.99) | 37 | 13.87 (7.57) | 38 | 13.37 (7.28) | 40 | 16.77 (9.26) | 42 |
| **HY-C3** | Baseline | 15.87 (13.85) | 46 | 19.48 (15.52) | 50 | 20.70 (15.05) | 48 | 18.06 (15.29) | 49 |
| **HY-C3** | Post | 15.15 (13.88) | 44 | 16.65 (13.63) | 48 | 17.29 (14.41) | 41 | 16.86 (15.73) | 47 |
| **HY-C3** | FU | 17.91 (14.71) | 37 | 14.58 (14.44) | 38 | 15.01 (13.48) | 40 | 17.36 (16.14) | 42 |
| **ADHD-C3** | Baseline | 7.14 (5.31) | 46 | 7.39 (5.53) | 47 | 7.42 (6.26) | 45 | 7.73 (5.55) | 49 |
| **ADHD-C3** | Post | 5.88 (5.51) | 41 | 5.24 (4.78) | 42 | 5.12 (5.68) | 40 | 7.75 (5.74) | 44 |
| **ADHD-C3** | FU | 6.56 (5.65) | 36 | 4.67 (4.95) | 38 | 4.61 (4.84) | 38 | 7.77 (6.06) | 40 |
| **MI-BRIEF** | Baseline | 90.96 (17.33) | 40 | 90.82 (16.94) | 44 | 89.21 (20.62) | 41 | 91.26 (20.07) | 45 |
| **MI-BRIEF** | Post | 84.93 (18.81) | 34 | 87.53 (16.76) | 44 | 84.05 (21.38) | 38 | 92.49 (17.86) | 38 |
| **MI-BRIEF** | FU | 83.38 (23.44) | 29 | 83.97 (18.90) | 34 | 80.81 (19.56) | 34 | 93.38 (23.06) | 37 |
| **BRI-BRIEF** | Baseline | 53.19 (13.92) | 43 | 53.04 (16.41) | 45 | 52.38 (16.20) | 44 | 53.57 (15.72) | 45 |
| **BRI-BRIEF** | Post | 51.00 (16.05) | 34 | 50.91 (14.77) | 45 | 50.85 (14.72) | 39 | 52.30 (15.29) | 38 |
| **BRI-BRIEF** | FU | 53.04 (15.06) | 28 | 48.99 (14.17) | 35 | 47.56 (13.63) | 36 | 52.82 (17.14) | 38 |
| **Note**. IN = Inattention scale; HY = Hyperactivity/impulsivity scale; BRI = Behaviour Regulation Index; MI = Metacognition Index; Post = Posttreatment; FU = 6-month Follow-up; n = Number of completed cases | | | | | | | | | |

| **Supplement Table S4b** Mean score for parent-ratings for complete cases per intervention, at baseline, posttreatment  and follow-up | | | | | | | | | |
| --- | --- | --- | --- | --- | --- | --- | --- | --- | --- |
|  |  | **Slow Cortical**  **Potential** | | **Live Z-Score** | | **Working Memory**  **Training** | | **Treatment as Usual** | |
| **Measure** | **Time** | **Mean (SD)** | **n** | **Mean (SD)** | **n** | **Mean (SD)** | **n** | **Mean (SD)** | **n** |
| **IN-C3** | Baseline | 18.57 (5.90) | 51 | 19.44 (5.43) | 49 | 20.07 (6.51) | 50 | 19.68 (5.63) | 49 |
| **IN-C3** | Post | 16.35 (5.97) | 48 | 18.10 (5.29) | 45 | 18.29 (7.61) | 46 | 19.28 (5.35) | 49 |
| **IN-C3** | FU | 16.17 (6.05) | 41 | 17.23 (5.42) | 44 | 16.77 (7.50) | 39 | 18.35 (5.57) | 48 |
| **HY-C3** | Baseline | 17.08 (11.24) | 51 | 18.92 (10.69) | 49 | 20.88 (11.67) | 50 | 16.39 (10.29) | 49 |
| **HY-C3** | Post | 15.47 (10.02) | 48 | 17.30 (10.53) | 45 | 20.02 (11.49) | 45 | 16.42 (10.11) | 49 |
| **HY-C3** | FU | 15.12 (10.88) | 41 | 16.55 (11.22) | 44 | 17.01 (11.85) | 39 | 15.95 (10.51) | 48 |
| **ADHD-C3** | Baseline | 9.69 (5.12) | 51 | 10.56 (4.71) | 50 | 10.85 (5.49) | 50 | 10.14 (4.74) | 49 |
| **ADHD-C3** | Post | 7.83 (4.90) | 48 | 8.95 (4.66) | 45 | 9.74 (5.67) | 45 | 9.73 (4.68) | 49 |
| **ADHD-C3** | FU | 7.95 (5.11) | 41 | 8.46 (5.64) | 44 | 8.21 (5.73) | 39 | 9.39 (5.08) | 47 |
| **MI-BRIEF** | Baseline | 103.50 (11.96) | 51 | 103.17 (11.37) | 50 | 104.20 (17.56) | 48 | 102.61 (13.75) | 49 |
| **MI-BRIEF** | Post | 98.78 (14.59) | 45 | 100.71 (12.67) | 45 | 101.75 (19.05) | 40 | 103.34 (13.78) | 48 |
| **MI-BRIEF** | FU | 95.37 (16.57) | 38 | 99.47 (13.85) | 37 | 98.25 (18.89) | 32 | 101.53 (14.73) | 47 |
| **BRI-BRIEF** | Baseline | 57.07 (12.32) | 51 | 53.35 (12.29) | 50 | 58.49 (13.71) | 49 | 54.46 (9.97) | 49 |
| **BRI-BRIEF** | Post | 55.80 (13.55) | 46 | 52.34 (13.07) | 45 | 55.89 (14.16) | 40 | 54.47 (11.57) | 48 |
| **BRI-BRIEF** | FU | 55.47 (14.18) | 38 | 51.91 (11.80) | 37 | 51.84 (13.51) | 32 | 53.15 (12.15) | 48 |
| **Note**. IN = Inattention scale; HY = Hyperactivity/impulsivity scale; BRI = Behaviour Regulation Index; MI = Metacognition Index; Post = Posttreatment; FU = 6-month Follow-up; n = Number of completed cases | | | | | | | | | |

| **Supplement Table S4c** Mean score for self-ratings for complete cases per intervention, for baseline, posttreatment  and follow-up | | | | | | | | | |
| --- | --- | --- | --- | --- | --- | --- | --- | --- | --- |
|  |  | **Slow Cortical**  **Potential** | | **Live Z-Score** | | **Working Memory**  **Training** | | **Treatment as Usual** | |
| **Measure** | **Time** | **Mean (SD)** | **n** | **Mean (SD)** | **n** | **Mean (SD)** | **n** | **Mean (SD)** | **n** |
| **IN-C3** | Baseline | 16.75 (7.33) | 51 | 15.42 (6.56) | 50 | 16.17 (7.31) | 51 | 15.10 (6.96) | 49 |
| **IN-C3** | Post | 15.62 (6.96) | 49 | 13.38 (6.22) | 48 | 15.26 (6.22) | 47 | 14.72 (6.46) | 48 |
| **IN-C3** | FU | 14.42 (6.62) | 44 | 13.04 (5.80) | 46 | 12.86 (6.21) | 42 | 14.57 (7.45) | 49 |
| **HY-C3** | Baseline | 16.36 (9.69) | 51 | 16.02 (8.43) | 50 | 16.99 (9.06) | 51 | 15.08 (7.32) | 49 |
| **HY-C3** | Post | 14.99 (9.94) | 49 | 14.43 (7.07) | 47 | 15.77 (8.75) | 47 | 13.76 (8.34) | 48 |
| **HY-C3** | FU | 14.41 (8.50) | 44 | 13.63 (7.62) | 46 | 15.27 (8.08) | 42 | 13.38 (7.60) | 49 |
| **ADHD-C3** | Baseline | 4.79 (3.97) | 51 | 4.08 (3.73) | 50 | 5.00 (4.04) | 51 | 4.40 (3.49) | 49 |
| **ADHD-C3** | Post | 3.91 (3.99) | 47 | 3.27 (3.53) | 44 | 4.06 (3.31) | 47 | 4.30 (3.59) | 47 |
| **ADHD-C3** | FU | 3.07 (3.40) | 41 | 2.76 (2.63) | 46 | 3.12 (2.71) | 42 | 3.73 (3.90) | 49 |
| **HRQoL-index** | Baseline | 39.06 (5.92) | 50 | 39.52 (5.46) | 50 | 38.96 (5.98) | 50 | 38.44 (5.35) | 48 |
| **HRQoL-index** | Post | 38.90 (5.82) | 49 | 40.58 (5.07) | 48 | 38.98 (6.35) | 46 | 38.55 (5.77) | 49 |
| **HRQoL-index** | FU | 40.32 (5.26) | 44 | 40.33 (5.14) | 46 | 39.46 (5.68) | 41 | 38.03 (6.06) | 49 |
| **Note**. IN = Inattention scale; HY = Hyperactivity/impulsivity scale; BRI = Behaviour Regulation Index; MI = Metacognition Index; HRQoL-index = Health-Related Quality of Life index from KIDSCREEN-27; Post = Posttreatment;  FU = 6-month Follow-up; n = Number of completed cases | | | | | | | | | |

| **Supplement Table S5** Overview of mean differentiation values for of participants last three sessions for feedback trials (mean diff FB) and transfer trials (mean diff TR) for each subject. | | | | | |
| --- | --- | --- | --- | --- | --- |
| **Subject** | **Mean Diff FB**  (in µV) | **Mean Diff TR**  (in µV) | **Subject** | **Mean Diff FB**  (in µV) | **Mean Diff TR**  (in µV) |
| **1** | 59,28 | 45,23 | **26^*^** | 24,60 | 16,78 |
| **2^*^** | 86,42 | 42,05 | **27** | -9,80 | 24,63 |
| **3** | 33,39 | 24,60 | **28** | 38,95 | 48,04 |
| **4** | -5,45 | -5,22 | **29** | 23,77 | 19,62 |
| **5** | 24,13 | -13,58 | **30** | -0,05 | 2,53 |
| **6** | 20,73 | -4,73 | **31** | -11,76 | -24,78 |
| **7** | 8,27 | -25,42 | **32** | 18,73 | -2,23 |
| **8** | 2,26 | -4,27 | **33^*^** | 12,45 | 31,30 |
| **9** | 8,18 | 0,38 | **34^*^** | 9,07 | 4,56 |
| **10** | -0,39 | -2,77 | **35** | 4,32 | -13,25 |
| **11^*^** | 44,16 | 36,57 | **36** | 19,76 | 3,93 |
| **12^*^** | -5,34 | 24,74 | **37** | -1,10 | -7,40 |
| **13^*,¤^** | 118,14 | 190,29 | **38** | 10,45 | 0,43 |
| **14^*^** | 54,10 | 11,25 | **39** | -2,71 | 3,20 |
| **15** | 9,79 | -2,75 | **40** | 21,38 | -15,01 |
| **16** | -8,02 | 20,87 | **41** | -11,77 | -6,75 |
| **17^*^** | 30,70 | 36,76 | **42** | 2,53 | -11,80 |
| **18** | 65,31 | 43,13 | **43** | 23,52 | 19,14 |
| **19** | -21,41 | 3,62 | **44^*^** | 36,04 | 42,50 |
| **20^*^** | 28,32 | 27,38 | **45** | -2,68 | 18,50 |
| **21** | -11,65 | 3,24 | **46** | -8,85 | 2,10 |
| **22** | -9,01 | -8,92 | **47** | 7,27 | 1,88 |
| **23^*^** | -1,78 | 11,73 | **48** | -8,01 | -7,82 |
| **24** | -0,06 | 2,61 | **49** | 14,99 | -10,87 |
| **25** | 17,09 | 1,89 | **Total** | 16,60 | 12,52 |
|  |  |  |  |  |  |
| *Positive value indicates correct differentiation as intended, while a negative value indicated differentiation in the opposite direction.*  **Note:^*^** = learners; ¤ = outlier | | | | | |
| 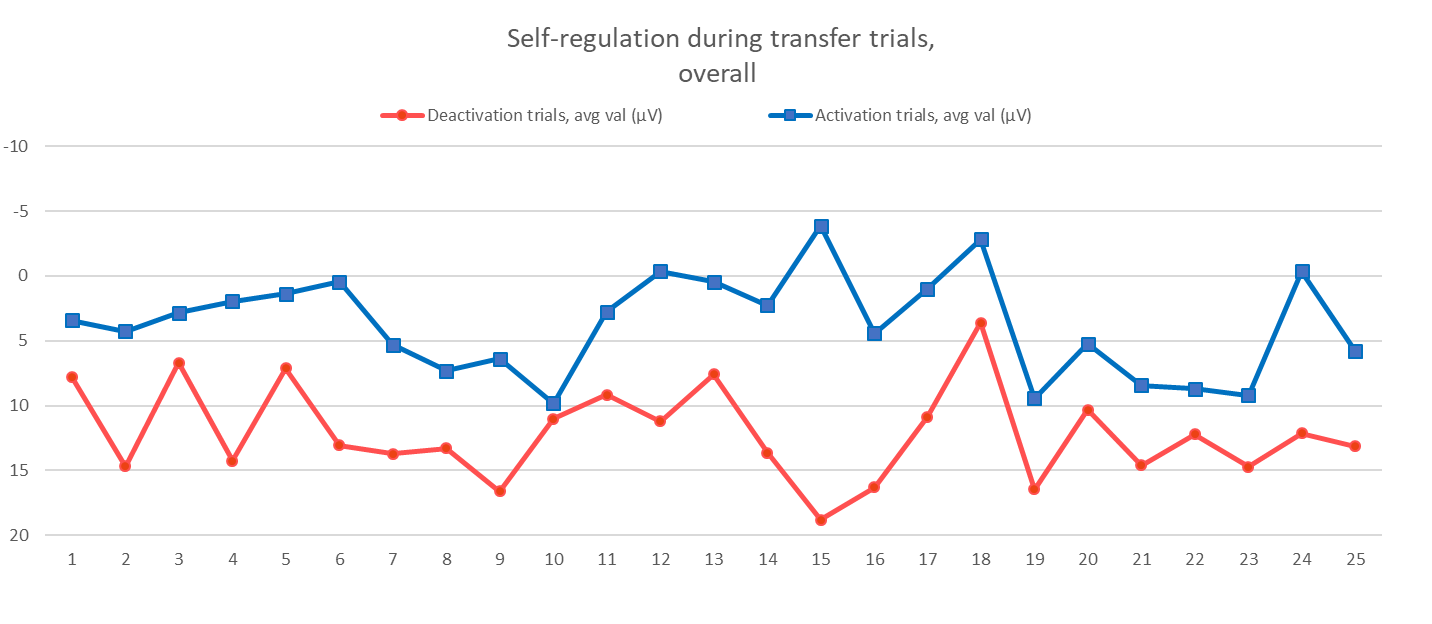 | | | | | |
| 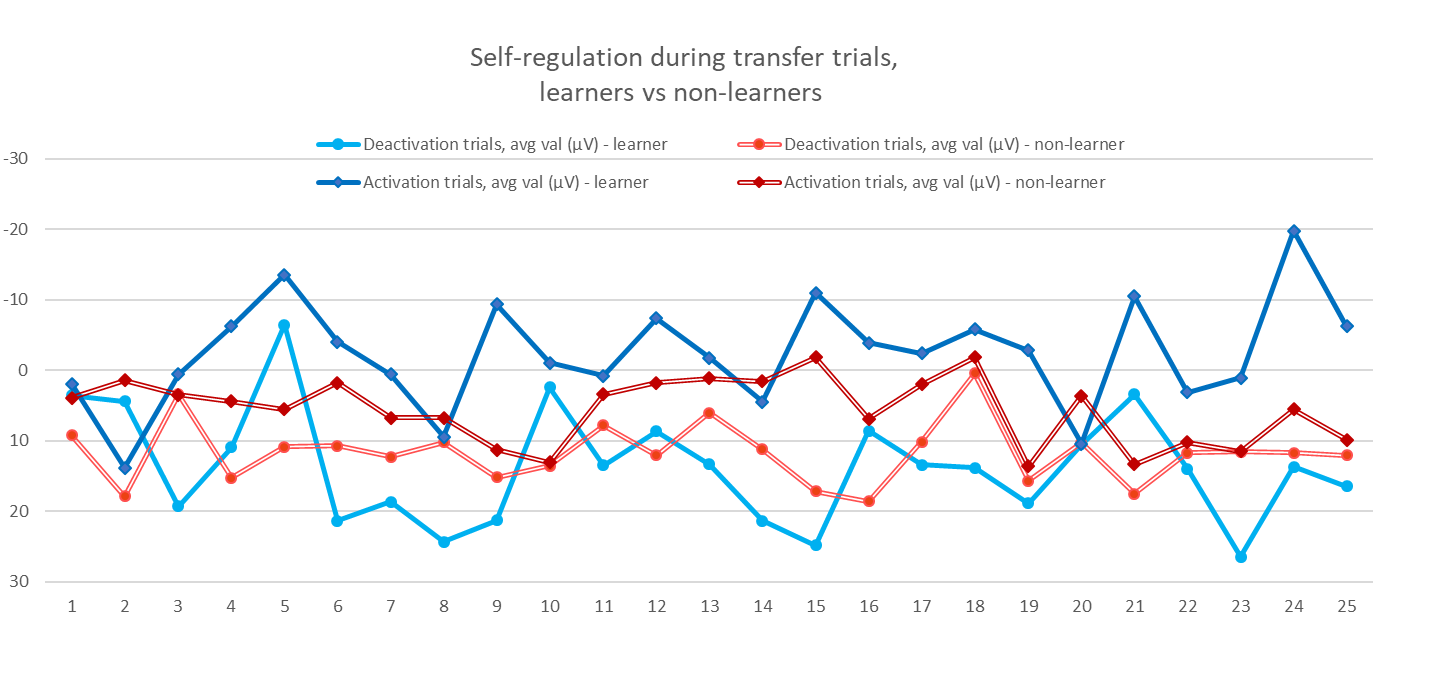 | | | | | |
| **Supplement Fig. S1** Self-regulation per session for slow cortical potential neurofeedback.  The top graph shows the average self-regulation during SCP transfer trials, for all participants per session. The lower graph illustrates the differences in self-regulation during SCP transfer trials, per session, between learners and non-learners. Participant 13 (outlier) is not included in the graphs. | | | | | |

| **Supplement Table S6** Slow Cortical Potential neurofeedback, learners versus non-learners from baseline to posttreatment and 6-month follow-up | | | | | | |
| --- | --- | --- | --- | --- | --- | --- |
|  | **Posttreatment** | | | **6-month follow-up** | | |
| **Measure (Rater)** | **Group difference in change score**  **(95% CI)** | **Sig.** | **Cohen’s d** | **Group difference in change score**  **(95% CI)** | **Sig.** | **Cohen’s d** |
| IN-C3 (T) | 2.68 (-0.61 to 5.97) | 0.108 | 0.38 | 4.20 (-0.14 to 8.53) | 0.057 | 0.59 |
| IN-C3 (P) | 0.43 (-2.68 to 3.55) | 0.782 | 0.07 | -1.42 (-4.77 to 1.92) | 0.398 | -0.24 |
| IN-C3 (S) | 1.11 (-2.41 to 4.62) | 0.529 | 0.16 | 1.05 (-3.19 to 5.28) | 0.622 | 0.15 |
| HY-C3 (T) | 2.03 (-3.26 to 7.32) | 0.442 | 0.15 | 0.80 (-6.32 to 7.93) | 0.822 | 0.06 |
| HY-C3 (P) | 0.47 (-3.05 to 3.99) | 0.790 | 0.04 | -0.92 (-5.86 to 4.02) | 0.710 | -0.08 |
| HY-C3 (S) | 2.60 (-0.70 to 5.90) | 0.119 | 0.27 | 2.66 (-1.03 to 6.35) | 0.155 | 0.28 |
| ADHD-C3 (T) | 1.80 (-0.51 to 4.10) | 0.123 | 0.34 | 1.03 (-2.30 to 4.35) | 0.536 | 0.19 |
| ADHD-C3 (P) | 1.75 (-0.91 to 4.41) | 0.192 | 0.35 | -0.69 (-3.64 to 2.27) | 0.643 | -0.14 |
| ADHD-C3 (S) | -1.14 (-3.27 to 0.99) | 0.285 | -0.29 | -0.69 (-2.98 to 1.60) | 0.550 | -0.18 |
| MI-BRIEF (T) | 11.03 (1.17 to 20.88) | 0.029* | 0.69 | -1.10 (-19.56 to 17.35) | 0.904 | -0.07 |
| MI-BRIEF (P) | 4.30 (-3.63 to 12.23) | 0.282 | 0.36 | 2.40 (-7.24 to 12.05) | 0.618 | 0.20 |
| BRI-BRIEF (T) | 7.93 (0.73 to 15.13) | 0.032* | 0.57 | -0.79 (-8.64 to 7.06) | 0.838 | -0.06 |
| BRI-BRIEF (P) | -1.11 (-6.09 to 3.86) | 0.655 | -0.09 | -0.73 (-7.19 to 5.73) | 0.820 | -0.06 |
| HRQoL-index (S) | -0.71 (-4.30 to 2.89) | 0.693 | -0.12 | 1.06 (-2.96 to 5.08) | 0.599 | 0.18 |
| **Note**: Positive numbers favor learners.  IN-C3 = Inattention subscale Conners-3; HY-C3 = Hyperactivity subscale Conners-3; ADHD-C3 = ADHD-index Conners-3; MI-BRIEF = Metacognition Index BRIEF; BRI-BRIEF = Behavioral Regulation Index BRIEF; HRQoL-index = Health-Related Quality of Life index from KIDSCREEN-27; T = Teacher; P = Parent; S = Self; * p. <=0.05 | | | | | | |

| **Supplement Table S7** Results for blindness questionnaire for teachers, sorted per intervention | | | | |
| --- | --- | --- | --- | --- |
|  | **SCP** | **LZS** | **WMT** | **TAU** |
| Teacher is aware of student’s participation in study: | 93% | 81% | 97% | 85% |
| Teacher selected correct intervention group: | 56% | 25% | 66% | 21% |
|  |  |  |  |  |
| **Reason for selecting group:** |  |  |  |  |
| Guessing: | 41% | 29% | 17% | 30% |
| Behavioral changes: | 33% | 16% | 31% | 9% |
| Info from student/parent: | 74% | 45% | 69% | 55% |
| **Note**: SCP = Slow Cortical Potential; LZS = Live Z-Score; WMT = Working Memory Training; TAU = Treatment-as-Usual | | | | |

| **Supplement Table S8** Number of participants reporting Adverse Events that increased to at least problematic, according to Pediatric Side Effects Checklist, by treatment group. | | | |
| --- | --- | --- | --- |
|  | **SCP (n=50)** | **LZS (n=50)** | **WMT (n=51)** |
| *Adverse Event^a^* | Baseline to Posttreatment (first two weeks of treatment) | Baseline to Posttreatment (first two weeks of treatment) | Baseline to Posttreatment (first two weeks of treatment) |
| **Restlessness** | 6 (3) | 9 (8) | 11 (8) |
| **Problems falling asleep** | 10 (8) | 5 (4) | 10 (8) |
| **Concentration problems** | 5 (5) | 6 (4) | 12 (8) |
| **Sleepiness** | 6 (3) | 7 (6) | 7 (6) |
| **Irritability** | 4 (2) | 8 (5) | 6 (4) |
| **Agitated** | 2 (1) | 7 (6) | 9 (7) |
| **Memory problems** | 6 (5) | 5 (3) | 6 (4) |
| **Tiredness** | 6 (5) | 5 (3) | 4 (4) |
| **Depression** | 5 (4) | 2 (2) | 5 (4) |
| **Loss of appetite** | 2 (2) | 5 (4) | 4 (4) |
| **Headaches** | 4 (3) | 5 (4) | 2 (2) |
| **Anxiety** | 2 (1) | 4 (4) | 5 (4) |
| **Stomach issues** | 3 (3) | 3 (2) | 4 (3) |
| **Anger** | 4 (2) | 5 (3) | 1 (0) |
| **Increased appetite** | 2 (2) | 4 (3) | 2 (1) |
| **Chills** | 2 (1) | 3 (1) | 3 (3) |
| **Increased thirst** | 2 (0) | 2 (0) | 3 (2) |
| **Nightmares / vivid dreams** | 4 (4) | 2 (1) | 0 (0) |
| **Nausea** | 1 (1) | 1 (1) | 2 (2) |
| **Dizziness** | 1 (1) | 0 (0) | 3 (3) |
| **Weight loss** | 3 (1) | 1 (1) | 0 (0) |
| **Constipation** | 1 (1) | 1 (1) | 1 (1) |
| **Diarrhea** | 2 (2) | 1 (1) | 0 (0) |
| **Tremors** | 1 (1) | 0 (0) | 2 (1) |
| **Dry mouth/ dry eyes** | 1 (0) | 1 (0) | 1 (1) |
| **Speech difficulties** | 0 (0) | 1 (1) | 1 (0) |
| **Increased sweating** | 0 (0) | 2 (2) | 0 (0) |
| **Panic attacks** | 0 (0) | 0 (0) | 2 (1) |
| **Rash** | 0 (0) | 2 (2) | 0 (0) |
| **Allergic reactions** | 0 (0) | 1 (1) | 1 (0) |
| **Muscle stiffness** | 0 (0) | 1 (1) | 0 (0) |
| **Stiffness in Jaw** | 0 (0) | 0 (0) | 1 (1) |
| **Blurred vision** | 0 (0) | 0 (0) | 1 (1) |
| **Increased salivation** | 0 (0) | 1 (1) | 0 (0) |
| **Weight gain** | 0 (0) | 1 (1) | 0 (0) |
| **Breast tension** | 0 (0) | 1 (0) | 0 (0) |
| **Palpitation** | 0 (0) | 1 (0) | 0 (0) |
| **Chest pain** | 0 (0) | 0 (0) | 1 (1) |
| **Acne** | 0 (0) | 0 (0) | 1 (0) |
| **Increase urine output** | 0 (0) | 0 (0) | 1 (0) |
| **Bed wetting** | 1 (1) | 0 (0) | 0 (0) |
| **Note**. SCP = Slow Cortical Potential; LZS = Live Z-Score; WMT = Working Memory Training; TAU = Treatment-as-Usual  ^a^ Translated from Swedish version | | | |
